# Facile synthesis 3D flexible core-shell graphene/glass fiber via chemical vapor deposition

**DOI:** 10.1186/1556-276X-9-394

**Published:** 2014-08-13

**Authors:** Cheng Yang, Yuanyuan Xu, Chao Zhang, Zhencui Sun, Chuansong Chen, Xiuhua Li, Shouzhen Jiang, Baoyuan Man

**Affiliations:** 1 College of Physics and Electronics, Shandong Normal University, Jinan 250014, People’s Republic of China; 2 Lishan College, Shandong Normal University, Jinan 250014, People’s Republic of China

**Keywords:** Flexible, Graphene core-shell structure, Two-heating reactor

## Abstract

Direct deposition of graphene layers on the flexible glass fiber surface to form the three-dimensional (3D) core-shell structures is offered using a two-heating reactor chemical vapor deposition system. The two-heating reactor is utilized to offer sufficient, well-proportioned floating C atoms and provide a facile way for low-temperature deposition. Graphene layers, which are controlled by changing the growth time, can be grown on the surface of wire-type glass fiber with the diameter from 30 nm to 120 um. The core-shell graphene/glass fiber deposition mechanism is proposed, suggesting that the 3D graphene films can be deposited on any proper wire-type substrates. These results open a facile way for direct and high-efficiency deposition of the transfer-free graphene layers on the low-temperature dielectric wire-type substrates.

**PACS:**

81.05.U-; 81.07.-b; 81.15.Gh

## Background

Graphene as typical sp^2^ hybridized carbon has been attracting extensive scientific interest from both experimental and theoretical communities in the recent years. Graphene has been reported by numerous papers on the growth [1-6], properties [7,8], and applications [9-11]. In most applications, such as supercapacitor, sensor [12], catalysis [13], battery [14], and water treatment applications [15], a small quantity of graphene is not sufficient; 2D graphene sheets with superior physical and electronic properties must be integrated into large-surface-area macroscopic three-dimensional (3D) carbon nanostructures [13-25]. Different carbon allotropes or complex compound structures, e.g., carbon nanotubes [13,15], carbon nanofibers [26], graphene networks [14,16,17,23], and carbon-based hybrid nanostructures [12,25], have been used to prepare the 3D nanostructured carbon materials. Several fabrication approaches such as chemical or thermal reduction of graphene oxide [17,18], hydrothermal carbonization [22], laser-based [27], and CVD [14] approach have been reported for the preparation of carbonaceous nanostructures. Graphene films or composites (reduced graphene oxide r-GO,) have been traditionally grown by chemical or thermal reduction of graphene oxide exfoliated from low-cost graphite [17,18]. The resulting r-GO, however, exhibits severely compromised conductivity due to the abundant defects, numerous non-ideal contacts between graphene sheets and functional moieties created during the synthesis procedures. In addition, this method is time-consuming due to the multi-step processes, including the high-temperature reduction process and a transfer process [24]. The performance of graphene-based supercapacitors, sensors, and other devices is seriously limited by such shortcomings. These problems can potentially be overcome by the macroscopic CVD graphene-based foam (GF) structures [14]. Three-dimensional architectures, with the continuous covalently bonded two-dimensional graphene building blocks, greatly reduce or eliminate the internal contact thermal resistance. The porous nature of this new-type 3D graphene material, with a large specific surface area (up to 850 m^2^ g^-1^) [14], is also suitable to make functional composites by filling the pores with nanoparticles, polymers, or other functional materials. However, the CVD graphene foam, which is formed on the nickel or copper foam, requires an etching processes to be transferred onto a foreign substrate. The process remains expensive and time-consuming [14,24,25]. Herein, we report a simple two-heating reactor CVD method for the direct formation of self-assembled flexible 3D core-shell graphene/glass fiber. This method presents us a promising transfer-free technique for fabrication 3D graphene nanostructures. Our new method involves a single-step, lower-temperature (600°C), yet its properties including the conductivity are comparable to those of CVD graphene foam.

## Methods

**Figure 1:**
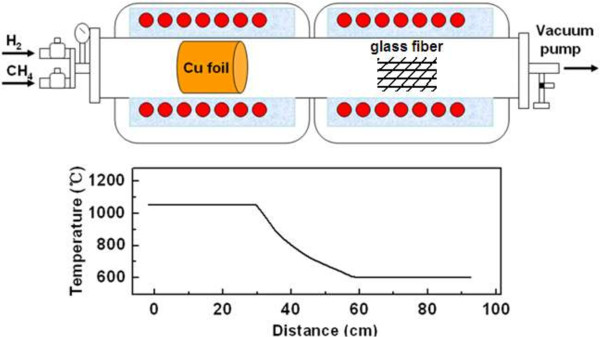
**Schematic diagram of the growth of 3D core-shell graphene/glass fiber.** By CVD using a two-heating reactor.

As shown in Figure 1, the experiments were performed in the furnace with two-heating reactor. An 8 × 10 cm^2^ strip of copper foils serving on the catalyst for the thermal dissociation of CH_4_ was located in higher constant-temperature zone (approximately 1,000°C), and the glass fiber membrane substrates (silica fiber, 25 mm in diameter and 49 um in depth) were spaced in the lower constant-temperature zone (600°C). Next, the horizontal quartz tube was pumped to 1.0 × 10^-6^ Torr and heated in the meanwhile. When the temperature reached 300°C, the Cu foil surrounding the tube was annealed in the flow of H_2_ and Ar (100 sccm/500 sccm) to remove the copper oxide. After another 30 min of annealing at 1,000°C, CH_4_ (50 sccm) and H_2_ (50 sccm) were introduced for 10 to 120 min of growth. Finally, the furnace was cooled down to the ambient temperature rapidly by simply opening the furnace.

Following growth, the morphology of the sample was characterized with scanning electron microscope (SEM, Zeiss Gemini Ultra-55, Carl Zeiss, Inc., Oberkochen, Germany) and transmission electron microscope (TEM, JEM-2100 F, JEOL Ltd., Akishima-shi, Japan). Raman spectra were obtained with a HORIBA HR800 Raman microscopy system (HORIBA, Kyoto, Japan) (laser wavelength 473 nm and laser spot size about 0.5 mm). The resistance of the sample was measured by depositing the silver electrode on the surface.

## Results and discussion

**Figure 2:**
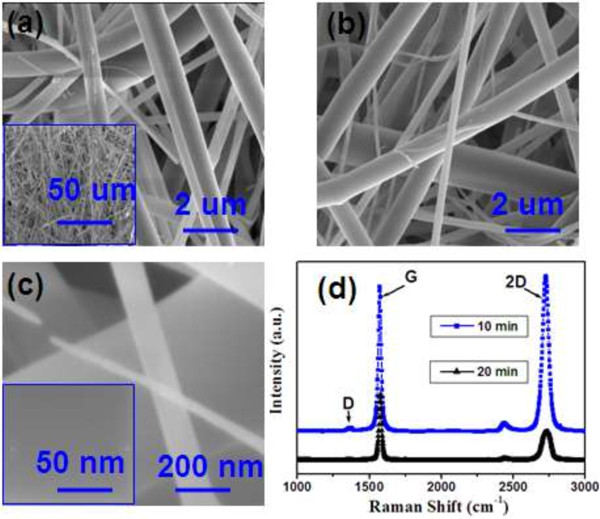
**SEM images before and after deposition and Raman spectra of the 3D graphene/glass fibers. (a) (b)** The SEM images before and after deposition of the graphene for 20 min on the glass fiber membrane surface. **(c)** SEM image of the 3D graphene/glass fibers with the different diameter. **(d)** Raman spectra of the 3D graphene/glass fibers deposited for 10 and 20 min.

Figure 2a,b exhibits the same magnification SEM images of the glass fiber membrane before and after the direct growth of the graphene films for 20 min. From Figure 2a and the inset, the membrane is formed by many wire-type glass fibers with the different diameter. A relatively uniform color is appreciated, and no rippled or wrinkled structures are detected on each glass fiber. The color difference between the glass fibers is caused by the imperfect focus mode due to the cylinder-shaped structure of the glass fiber. Typical SEM images of the glass fiber after the CVD deposition (Figure 2b) also give us persuasive and striking evidence of the uniform structure of the prepared graphene film. Figure 2b,c shows SEM images of the prepared sample under a different magnification factor. It is clear that the graphene film still possesses a uniform structure even under a high magnification (Figure 2c and the inset). It should be stressed that the graphene films can be grown on the surface of every wire-type glass fiber with the diameter from 30 nm to 2 um. Figure 2c shows the SEM images of the 3D core-shell graphene/glass fibers with the diameter of 30, 120, and 500 nm. We believed that there are no differences for the formation of 3D core-shell graphene/glass fibers on the different diameter glass wires, while the growth time is important for the synthesis of the 3D core-shell graphene/glass fibers. Different graphene layers can be formed on the glass wire results from the different growth time. Raman spectra (shown in Figure 2d) were performed to detect and characterize the graphene layers in the surface of the glass wires with the different growth times (10 and 20 min). Both of the Raman spectra present typical characteristics of graphene layers: obvious D, G, and 2D bands at approximately 1,340, approximately 1,588 and approximately 2,700 cm^-1^. For the Raman spectrum of the sample grown for 10 min, The *I*_2D_/*I*_G_ intensity ratio is approximately 1.1, and the full width at half maximum (FWHM) of 2D band is approximately 45 cm^-1^, which represents one to two layers of graphene film [28]. For the Raman spectrum of the sample grown for 20 min, the *I*_2D_/*I*_G_ intensity ratio is approximately 0.5, and the full width at half maximum (FWHM) of 2D band is approximately 55 cm^-1^, which represents three to five layers of graphene film [28]. Compared with the Raman spectrum of the monolayer graphene [1], the 2D band of the multilayer graphene is broader and can be fitted into multipeaks, which can be explained by the double-resonance theory: the electronic band structure and electron-phonon interactions change with the number of the graphene layers [29]. In particular, the observed small D band intensity as compared to the G band intensity indicated low levels of defects or local disorder in our MLG films.

**Figure 3:**
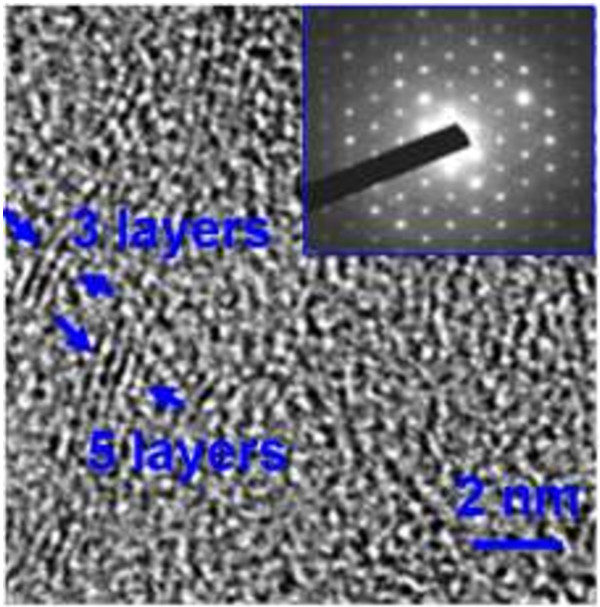
**HR-TEM image of the graphene layers deposited for 20 min.** The inset shows the ED pattern of the multi-layer graphene film.

It is possible to investigate the state of the graphene by transferring it to a small holey copper grid using TEM. The HR-TEM image (shown in Figure 3) was conducted on the sample to identify the number of the graphene layer. The edge-on image of graphene in Figure 3 indicates the thickness of the prepared graphene is three to five layers and the measured intergraphene spacing is approximately 0.34 nm, which is consistent with the previous report [28]. In addition, the electron diffraction (ED) pattern on the multilayer graphene film (the inset of Figure 3) reveals a hexagonal pattern, confirming the threefold symmetry of the arrangement of carbon atoms in graphene. These TEM results show direct evidence that multilayer graphene film is directly fabricated on the glass fibers.

In our experiment, the lower-temperature (600°C) growth is necessary due to the lower melting point of the glass fiber, which can be obtained by the revised CVD system with the two-heating reactor. The mechanism of synthesis of core-shell graphene/glass fiber structures by using such revised CVD system has been discussed here. The higher constant-temperature zone offers enough power for the dissociation of methane with the assist of copper catalyst, and the lower constant-temperature zone makes that the decomposed carbon atoms deposit readily on the substrate. Meanwhile, the distinct form of the temperature variation effectively controls the regions that the evaporated Cu atoms and the decomposed carbon atoms deposit. The gently declined temperature zone can make the evaporated Cu atoms deposit on the region close to the higher constant-temperature zone [30]. Higher-temperature (approximately 1,050°C) zone is required in our experiment. As is well known, thermal dissociation of methane is facile at temperatures above 1,000°C, and it is hard to proceed at the low temperature below 600°Χ, even though Cu catalyst is presented. The copper foil is used here to catalyze the methane thermal dissociation.

**Figure 4:**
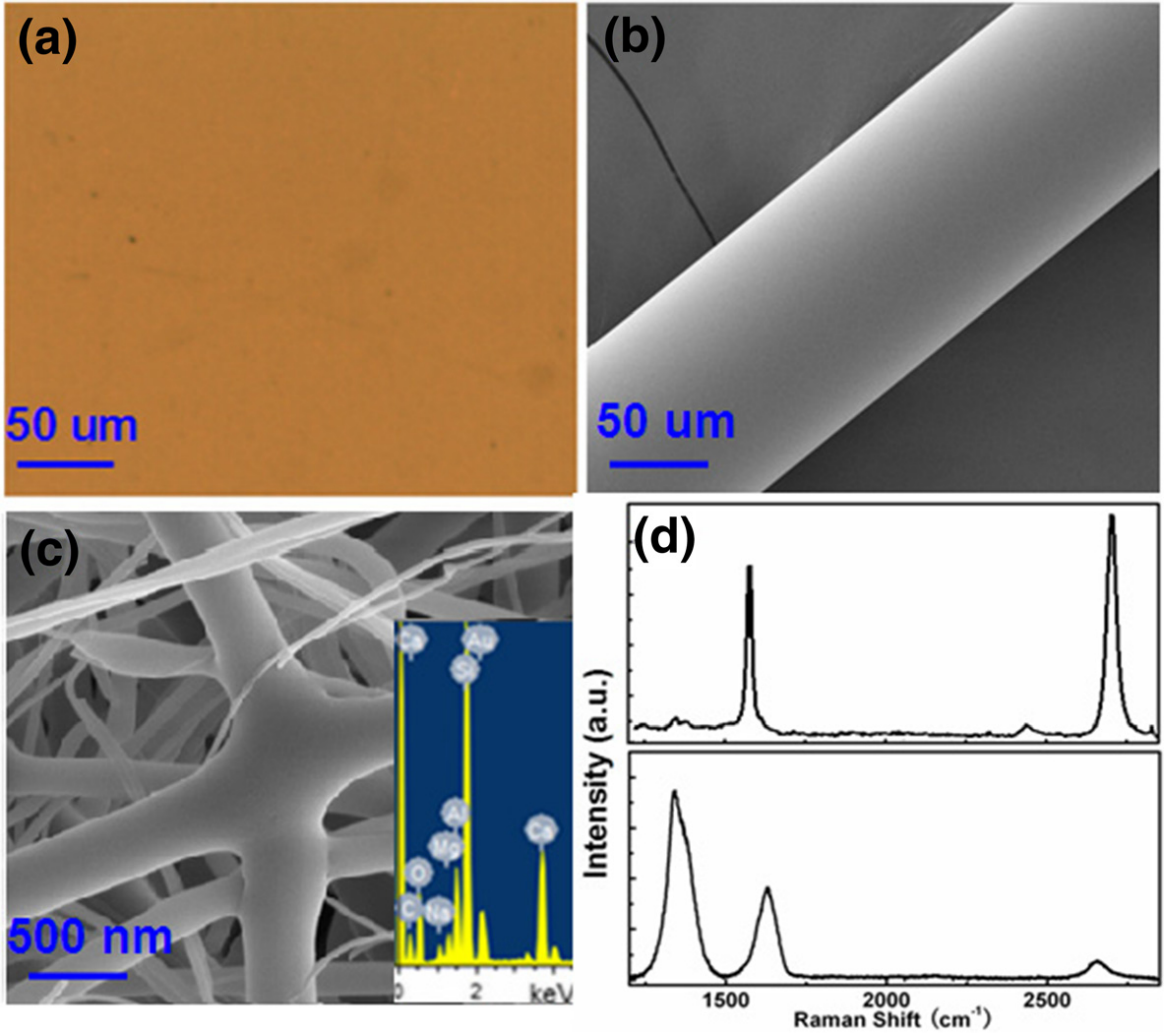
**SEM images of graphene films and Raman spectra. (a) (b) (c)** SEM images of graphene films grown for 120 min on the plant SiO_2_**(a)**, SMF **(b)** and glass fiber **(c)**. **(d)** Raman spectra obtained from the plant SiO_2_ substrate (upper) and glass fibers (lower).

It should be stressed that the graphene film can be grown on the plant SiO_2_ wafer and wire-type fiber substrates, while the grown graphene layers are different, especially after a longer time growth. The plant SiO_2_ wafer substrate and single-mode fiber (SMF, diameter is approximately 125 um, treated with acetone and deionized water to remove the opaque cover) are also used here to deposit graphene film for 120 min in the same CVD process. Figure 4a,b,c shows the SEM images of graphene films grown for 120 min on the plant SiO2, SMF, and glass fiber. A relatively uniform color is also appreciated and no rippled or wrinkled structures are detected on each substrate. Obvious D, G, and 2D bands at approximately 1,340, approximately 1,588 and approximately 2,700 cm^-1^ are also observed in both of the Raman spectra (shown in Figure 4d), which represents typical characteristics of graphene film. The upper spectrum in Figure 4d is obtained from the plant SiO_2_ substrate, which represents typical characteristics of monolayer graphene. The lower spectrum in Figure 4d is obtained from the glass fibers. The spectrum of the SMF (not shown here) is similar with that of the glass fiber. A disordered D band, located around 1,350 cm^-1^, and an active graphite G band, located around 1,600 cm^-1^, were observed. The spectrum of monolayer graphene on plant substrate is essentially the superimposition of that of multilayered graphene on the glass fibers, except the appearance of a larger D band and right shift of G band (likely arising from the defects introduced in the formation of the multilayered graphene [12]). At the same time, 2D band (approximately 2,680 cm^-1^) related to a graphene layer structure is also hardly observed. The *I*_2D_/*I*_G_ intensity ratio is only 0.2, and the full width at half maximum (FWHM) of 2D band is up to approximately 70 cm^-1^. These results represent that there are many graphene layers and many defects are formed on the wire-type glass fibers.

In fact, graphene growth on the plant SiO_2_ substrate are predominantly monolayer, due to the growth process is self-limited. As is well known, SiO_2_ has higher surface energy than after it is covered by graphene. Namely, the cohesion energy between SiO_2_ and graphene is higher than that of graphene-to-graphene. Therefore, after being covered by a layer of graphene, the carbon species become hard to nucleate on the graphene-covered area due to the relatively weak cohesion energy, refusing to form the second layer [31]. But, one exception occurs at the defects where the dangling bonds give more opportunities for carbon adsorption to form the multilayer or many-layered graphene. For the glass fiber case, there are many overlaps and defects on the surface. From the EDX spectrum (shown in the inset of Figure 4c), there are also many metal element existed in the SiO_2_ wires. The metal elements existed in the SiO_2_ wires are caused by the formation of the glass membranes. All of the overlaps and defects can be used as the catalyst sites to further grow the graphene layers. From Figure 4c, many graphene layers have been covered on the overlaps of the glass fibers, which revealed that carbon species are easily nucleate on such areas.

We also measured the sheet resistance (Rs) of the prepared graphene film obtained at room temperature. The calculated average value of the Rs is approximately 700, 300, and 180 Ω/sq for the plant SiO_2_, SMF, and glass fiber membrane substrate. The excellent electrical properties further demonstrate that high-quality graphene layers can be prepared using such two-heating reactor CVD system in the relatively low temperature. The lower sheet resistance of the glass fiber membrane samples is caused by the more layers of the graphene films.

## Conclusions

We have demonstrated the facile low-temperature growth of 3D graphene/glass fiber wire-type structures using a two-heating reactor. The higher constant-temperature zone offers enough power for the dissociation of methane with the assist of copper catalyst, and the lower constant-temperature zone makes that the decomposed carbon atoms deposit readily on the substrate. Graphene layers can be grown on the different diameter wire-type glass fiber surface to form graphene/glass fiber wire-type structures. The morphology and electrical properties of such structures can be controlled by changing the growth time. These results suggest that the 3D graphene films can be deposited on any proper wire-type substrates.

## Competing interests

The authors declare that they have no competing interests.

## Authors' contributions

CY and BM are the corresponding authors and designed the experiments and sample preparations and drafted the manuscript. YX, CZ, ZS, CC, XL, and SJ took part in the sample preparation and characterizations and discussed the results. All authors have read and approved the final manuscript.

## Authors' information

BM is a professor in the college of Physics and Electronics at Shandong Normal University, China. He is a Ph.D. supervisor. His main research interests include nanomaterials and laser plasma. CY has graduated from SungKyunKwan University (SKKU), Korea. Currently, he works at Shandong Normal University. His research subject is nanomaterials and their applications. YY, CZ, and ZS are currently doing their Ph.D. at Shandong Normal University. Their research subjects are related to 2D nanomaterials such as graphene, Bi_2_Se_3_, and MoS_2._ XL works in Lishan College at Shandong Normal University; her research focus is solar materials. SJ and CC are professors in the College of Physics and Electronics at Shandong Normal University. They are M.S. Supervisor. Their main interests include nanomaterials, mode-locked lasers, and laser plasma.
